# Biotin-Functionalized Semiconducting Polymer in an Organic Field Effect Transistor and Application as a Biosensor

**DOI:** 10.3390/s120811238

**Published:** 2012-08-13

**Authors:** Zin-Sig Kim, Sang Chul Lim, Seong Hyun Kim, Yong Suk Yang, Do-Hoon Hwang

**Affiliations:** 1 Electronics and Telecommunications Research Institute (ETRI), 218 Gajeongno, Yuseong-Gu, Daejeon 305-700, Korea; E-Mails: lsc@etri.re.kr (S.C.L.); kimsh@etri.re.kr (S.H.K.); jullios@etri.re.kr (Y.S.Y.); 2 Chemistry Institute for Functional Materials, Department of Chemistry, Pusan National University, Busan 609-735, Korea; E-Mail: dohoonhwang@pusan.ac.kr

**Keywords:** biosensor, F8T2, organic semiconductor, fluorescence, OTFT, biotin, avidin

## Abstract

This report presents biotin-functionalized semiconducting polymers that are based on fluorene and bithiophene co-polymers (F8T2). Also presented is the application of these polymers to an organic thin film transistor used as a biosensor. The side chains of fluorene were partially biotinylated after the esterification of the biotin with corresponding alcohol-groups at the side chain in F8T2. Their properties as an organic semiconductor were tested using an organic thin film transistor (OTFT) and were found to show typical *p*-type semiconductor curves. The functionality of this biosensor in the sensing of biologically active molecules such as avidin in comparison with bovine serum albumin (BSA) was established through a selective decrease in the conductivity of the transistor, as measured with a device that was developed by the authors. Changes to the optical properties of this polymer were also measured through the change in the color of the UV-fluorescence before and after a reaction with avidin or BSA.

## 1. Introduction

Since the pioneering reports of field-effect transistors (FETs) based on polymer and small organic molecule semiconductors were published, interest in this field has risen steadily for both technological and scientific reasons [1–12]. In recent years the number of publications on organic and polymer transistors has increased considerably. What makes organic FETs technologically interesting is their use as the main component in cheap and flexible electronic circuits. Major possible applications are radio frequency identification (RFID) tags, smart windows, e-paper and flexible displays [13–19]. Prototypes of these products have been demonstrated and are now getting close to being brought to the market [20]. Scientifically, organic semiconductors show interesting characteristics both similar to and different from known inorganic semiconductors, in particular amorphous silicon [21]. The charge transport and emission properties of organic semiconductors have been under intense investigation for years, and field-effect transistors have proven to be powerful tools for those efforts. Among many other advantages of organic thin film transistors (OTFTs), the fabrication of thin film transistor (TFT) by solution-based process is possible. Some examples of these are spin-coating, inkjet printing and gravure printing, which can enable the production of low-cost TFTs on a large-area substrate [20].

Recently, because they can be fabricated at low-cost, transported and disposed of easily, organic semiconductor materials have drawn more attention in the field of developing (FETs) for usage, not only in industrial devices such as chemical sensors or gas sensors, but also in medical devices, such as glucose sensors [22]. Therefore organic thin film transistors are an excellent alternative for use in disposable sensors. The usage of organic semiconductors as biosensors has become in recent years more enticing, because their structure can be changed in response to biologically relevant substances and such changes cause a decrease of conductivity of organic semiconductors [6,23]. These changes can be measured as electric signals through common detecting devices. Here we report on the characteristic properties of thin film transistors using organic semiconductor polymers and the functionalities of their organic field effect transistors as biosensors for the selective detection of biologically active proteins, for example, avidin *vs.* bovine serum albumin (BSA). Biotin binds extremely strongly to avidin or streptavidin; this binding interaction is widely applied to bioanalytical usages, such as the detection of biomolecules, clinical diagnosis and immunoassays [6,7,24–31].

Some OTFT-based DNA sensing was reported by Bartic *et al.*, in which they used ion-sensitive field effect transistors (ISFET) [32]. In the structure of ISFET, a metal gate was used as the sensitive layer. DNA molecules were immobilized on the metal gate. The superimposed gate voltage shifts the saturation current of DNA molecules. Because of the low mobility of organic semi-conductors, this method requires a high working voltage in comparison with that used in silicon-based ISFETs. When the organic semiconductor materials are used as the sensitive layer for detecting biologically active species, such as biomarkers, proteins, DNA or enzymes, they can provide high sensitivity, because the characteristics of the active organic semiconductor layer can be changed simultaneously [33]. In these characteristics are included mobility, threshold voltage, crystalline structure and conjugation length; all these influence the saturation current shift.

In this respect, the conjugated organic polymers with biotinylated side chains are going to bind to avidin with strong interactions, which will cause changes of the structure of the conjugated organic polymers. This means that in a conjugated conducting polymer semi-conductor, the length of the conjugated double bonds of the organic semiconductors, whose biotinylated side chains are bound to avidin, can be shorter than the length of those that are not bound [23]. These changes can be measured not only as changes of electrical signals, but also as changes in the color of UV-fluorescence. Previously, we reported some results of our research about organic thin film transistors in *ETRI Journal* [34]. In this report, we present some organic thin film transistors in which the conjugated organic semiconductor was used as the sensitive layer; we also describe a novel application of organic thin film transistors with organic semiconductors as the active sensitizing layer for the selective detection of biologically active species. This can be used as a biosensor.

## 2. Experimental Section

### 2.1. Synthesis of Biotinylated Semiconductor Polymer (F8T2)

**Figure 1.**
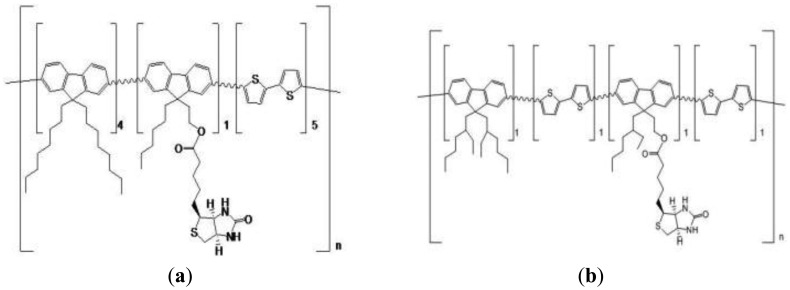
Two types of biotinylated organic semiconductor polymers (F8T2). (**a**) 10% and (**b**) 25% of the total side chains were biotinylated.

To fabricate the OTFT, we used a highly conductive Si wafer (resistivity 5–10 Ω·cm) as both a substrate and a gate electrode. The gate dielectric layer for all the devices was a thermally grown 300 nm thick SiO_2_ layer (capacitance per unit area of *C*o = 10 nF/cm^2^). For the source and drain electrodes, we fabricated an 80 nm thick Au layer with a 20 nm thick Cr adhesion layer. The biotinylated semiconductor polymers, which were used for preparing the devices in this report, are based on fluorene and bithiophene co-polymer, poly(9,9-dioctylfluorene-co-bithiophene) alternating copolymer (F8T2) [35,36]. The side chains of F8T2 were partially biotinylated through esterification of biotin with alcohol on the side chain of F8T2, which we synthesized (Figure 1).

The synthesis of the biotinylated F8T2 polymer was completed according to the Suzuki coupling method [37]. The two biotinylated F8T2 copolymers are shown in Figure 1. Figure 1(a) shows the biotinylated F8T2-copolymer, for which 10% of the total side chains were substituted with biotinylated side chains; Figure 1(b) shows the 25% of the total side chains biotinylated F8T2-copolymer. The biotinylation of the side chains of F8T2-copolymer succeeded according to the well known esterification of the alcohol groups with the biotin. The synthesized biotinylated final products were purified and the structures of the biotinylated F8T2 conducting polymers were analyzed. Our recent report describes the synthetic process of biotinylated F8T2-copolymers in detail [34].

### 2.2. Avidin and Bovine Serum Albumin (BSA) Proteins

To investigate an avidin-biotin based sensing system, we used two types of protein buffer solutions. One of these was BSA solution as standard protein solution, the other was avidin solution as specific protein solution. To prepare these two protein solutions, we used the commercially available proteins of avidin (Sigma-Aldrich) and BSA (Sigma-Aldrich). Both of these were dissolved in deionized water (DI water) or in a corresponding buffer solution and diluted to the corresponding concentration directly before the consumption of the solution. The concentration of the undiluted BSA-solutions in our experiments was 1 mg/mL, which means 1.5 × 10^'2^ M. The concentration of avidin-solutions was also 1 mg/mL and which means 1.6 × 10^'5^ M. After the dilution, the concentrations of the BSA and avidin solution were 8 × 10^'7^ M and 6 × 10^'7^ M, respectively.

### 2.3. Fabrication of Organic TFT Devices

**Figure 2.**
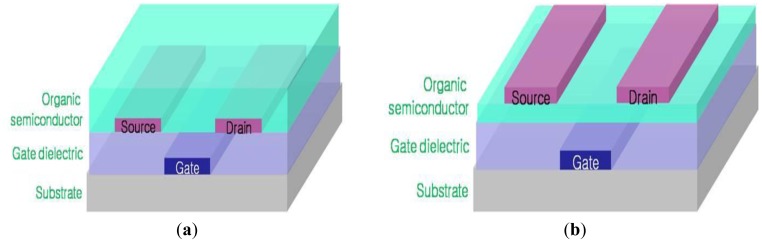
Schematic cross section of OTFT structures. (**a**) Bottom-contact and (**b**) Top-contact OTFT

For the fabrication of organic thin film transistor devices, the biotinylated F8T2 polymer was dissolved in the organic solvent *p*-xylene (0.04 wt.%) and the solution was filtered through a 0.45 μm syringe filter onto the previously prepared bottom-gated substrates, where it was conventionally spin-coated at 1,500 rpm for 30 seconds. After drying the solvent, the prepared devices were annealed on the hot-plate under N_2_ atmosphere at 250 °C for 1 h. A simplified cross-section diagram of the OTFT devices is shown in Figure 2. The surface of the fabricated OTFT devices with biotinylated F8T2 polymer was very hydrophobic and it was difficult to have it contact the water based solution of the detecting materials, such as the BSA or avidin solution. To improve the hydrophilicity of the surface of the biotinylated F8T2 polymer fabricated OTFT devices, those surfaces were treated with UV under O_3_ atmosphere for 10 min. After UV-treatment, the surfaces of the OTFT devices were more hydrophilic than they were before UV-treatment. Through contact angle measuring we found the optimal condition of UV-treatment for better hydrophilicity of the biotinylated F8T2 polymer OTFT devices. Before UV-treatment, the contact angle for water was 80° but after 10 min. UV-treatment it decreased down to 12°.

### 2.4. Measurement of the Electric Properties

The electric properties of the biotinylated F8T2 TFTs before and after exposure to the solutions containing the avidin were measured at room temperature using a Hewlett-Packard 4145B semiconductor parameter analyzer. For measurement of the electric properties of the fabricated devices, the F8T2 surface of each device was made to contact previously prepared BSA or avidin solutions for a reaction time of 10 min.; these devices were washed with deionized water and dried with N_2_ gas. As a reference device, one of the prepared organic TFT devices was treated only with DI water without any treatment of BSA or avidin solution.

## 3. Results and Discussion

### 3.1. OFET Characteristics

**Figure 3.**
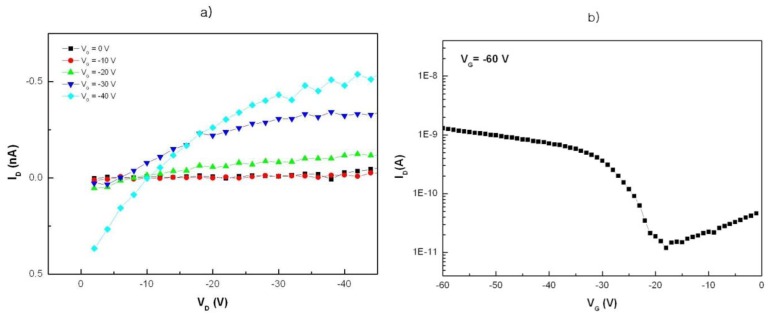
The typical plot of drain current I_D_ versus gate voltage V_G_ for biotinylated F8T2 TFTs. (**a**) Typical plot of the drain current I_D_ versus gate voltage V_G_ between 0 and '40 V. and (**b**) The On/Off-curve of the organic semiconductor field effect transistor (organic FET).

The fabricated OTFT devices in this research were characterized by generally accepted methods. According to the cross-sectional SEM image (not shown in this report), the thickness of the biotinylated F8T2 films was approximately 4,300 Å [31]. After annealing at 250 °C for 1 h under N_2_ atmosphere, the OTFT devices showed the typical I-V-curves of OTFTs. The V_G_ is between 0 V and '40 V. Figure 3 shows the typical plots of drain current I_D_ versus gate voltage V_G_ at a drain voltage V_D_ of '60 V for biotinylated F8T2 OTFTs, which were fabricated with a channel length of 10 μm, channel width of 100 μm, SiO_2_ gate dielectric thickness of 300 nm, and the On/Off-curve of the organic semiconductor field effect transistor (organic FET). Fabrication of the OTFT device was completed before exposing the compound to detecting materials, for example, BSA or avidin.

When the gate electrode was biased negatively with respect to the grounded source electrode, the OTFTs operated in accumulation mode, and the accumulated charges were holes. The field-effect mobility of the OTFT devices with biotinylated F8T2 was approximately 1.4 × 10^'5^ cm^2^/V·s and the on/off ratio was about 10^2^ (Figure 3).

The OTFT properties of these devices were slightly different from those of OTFT devices with normal F8T2, because, with the biotinylated F8T2, the side chains of the normal F8T2 were partially substituted with biotin functional groups, which interact with each other. The normal F8T2 semiconductor polymer consists of an alkyl-group as side chain, but the biotinylated F8T2 consists of not only the alkyl-group but also a biotin-functional group at the side chain. These biotin-groups interact with each other and also with the normal alkyl-group. These interactions can influence the semiconductor properties of the biotinylated F8T2, and can cause decreasing conductivity of the biotinylated F8T2 semiconductor.

### 3.2. Biosensor Characteristics

The fabricated OTFT devices using biotinylated F8T2 were also investigated as a biosensor. For this purpose, three kinds of device were prepared after annealing at 225 °C. The first of them was treated only with DI water, for a non-treated sample; the second was treated with standard protein solution, BSA; the third was treated with avidin solution as described. The treatment of the OTFT devices proceeded as follows. The devices were contacted with the protein-containing solution for 10 min. at room temperature. The concentrations of BSA and avidin aqueous solution were 8 × 10^'7^ M for avidin and 6 × 10^'7^ M for BSA. After 10 min. of contact time, each of the samples were rinsed with deionized water and dried with nitrogen gas. The electric properties of samples fabricated with biotinylated F8T2 were measured with the earlier described Hewlett-Packard 4145B semiconductor parameter analyzer.

**Figure 4.**
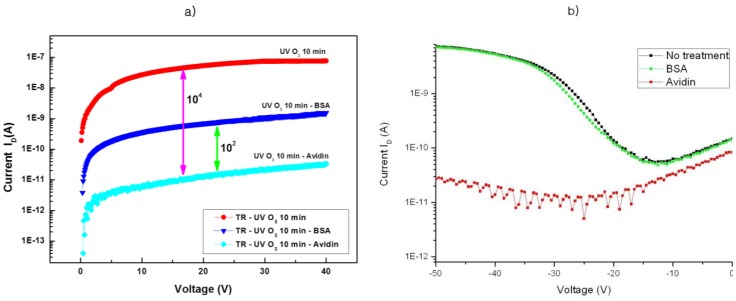
Changes of the characteristics of OTFT by detection of sensing materials. (**a**) Typical plot of drain current I_D_ versus gate voltage V_G_ between 0 and 40 V for biotinylated F8T2 TFTs. (**b**) On/Off-curve of the organic semiconductor field effect transistor (organic FET) before and after treatment with BSA or avidin.

The biotin functional group interacts extremely strongly with specific proteins, for example, avidin or streptavidin, but has almost no interaction with a standard protein, for example, BSA. The biotin-avidin combination is widely applied in the field of bioactive material detection and the applications of their combination are already published [24]. The weak interaction of biotin-BSA combination caused a slight decrease of the drain current and the conductivity of the sensing devices, but the strong interaction of the biotin-avidin combination caused a remarkable decrease in the drain current and also the conductivity of the sensing devices (Figure 4). In our experiments, three kinds of test devices were prepared. One of them was used as a reference for a blank test. The other two were used for testing BSA- or avidin-solution. All three of these devices were treated with 365 nm UV light for 10 min. under an air atmosphere, before the treatment with test-solution. The first one of them was treated with only DI water after UV O_3_ treatment, which is indicated as “UV O_3_ 10 min” in Figure 4(a). The second one of them was treated with BSA test-solution after UV O_3_ treatment, which is indicated as “UV O_3_ 10 min-BSA” in Figure 4(a). The third one of them was treated with avidin test-solution after UV O_3_ treatment, which is indicated as “UV O_3_ 10 min-Avidin” in Figure 4(a). After treatment with three kinds of test solution, the OTFT properties of all three devices were measured. The first device, which was treated with only DI water, showed higher conductivity than the other two devices (“UV O_3_ 10 min”) in Figure 4(a). The second device, which was treated with BSA test solution, showed a 2 order scale decrease in conductivity relative to the first one (“UV O_3_ 10 min-BSA”) in Figure 4(a). The third device, which was treated with avidin test solution, showed a four order scale decrease in conductivity relative to the first one (“UV O_3_ 10 min-Avidin”) in Figure 4(a). As Figure 4(a) shows, the conductivity of the device treated with avidin-solution decreased two orders more than that of the device treated with BSA-solution. Nevertheless, the conductivity of these two devices decreased below that of the device treated with DI-water. The difference of the drain current between avidin-treated and non-treated devices was much more significant than that between the BSA-treated and non-treated devices. After the exposure of OTFT devices to avidin solution, the decrease of the drain current and also the conductivity were so intense that the devices indicated no more OTFT properties, which meant a disappearance of semiconductor properties of biotinylated F8T2 (Figure 4(b)). In comparison to this result, the device treated with BSA-solution indicated the typical OTFT properties after exposure to the BSA-solution. These results indicate that the conductivity of the device treated with the BSA-solution decreased, but the semiconductor properties remained unchanged. The loss of the semiconductor properties of the biotinylated F8T2 can be explained by the shortening of the conjugation length of the carbon-carbon bond in biotinylated F8T2 polymer backbone, due to the strong interaction between biotin and avidin. The biotinylated F8T2 polymer semiconductor seemed to be useful for OTFTs and may be applied to developing an OTFT biosensor or FET for biosensor (BioFET).

**Figure 5.**
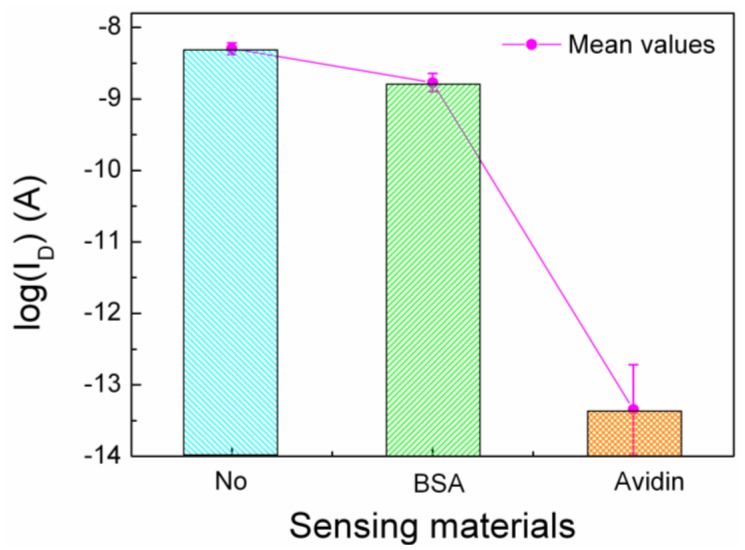
Changes of the drain current of OTFT by the detection of sensing materials. (No): after treatment with DI water. (BSA): after treatment with BSA-solution. (Avidin): after treatment with avidin-solution.

Changes of the drain current of OTFT with biotinylated F8T2 by detection of sensing materials are shown in Figure 5. The drain current of the OTFT device after treatment with only DI water is indicated as “No” in Figure 5. The drain current of the OTFT device after treatment with BSA solution is indicated as “BSA”, and it decreased slightly. The drain current of the OTFT device after treatment with the avidin solution is indicated as “Avidin”, and it decreased significantly by a five order scale. In Figure 5, the mean values of the drain current of 30 samples of OTFT devices for each sensing material are indicated. The decrease of drain current after avidin treatment lay far beyond the error bar of the measurement.

**Figure 6.**
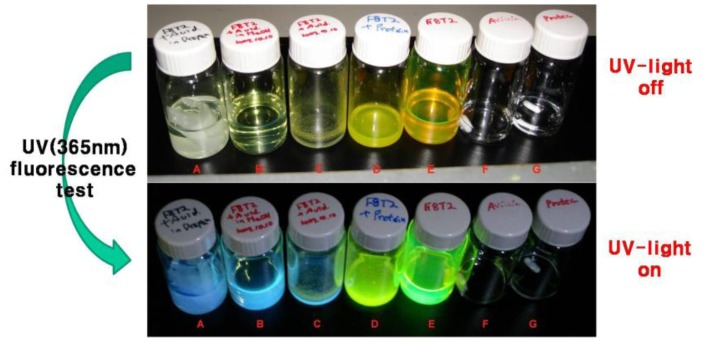
UV (365 nm) fluorescence test with BSA- or avidin-treated biotinylated F8T2. (**A**) biotinylated F8T2 with Avidin in dioxane. (**B**,**C**) biotinylated F8T2 with Avidin in MeOH. (**D**) biotinylated F8T2 with BSA in MeOH. (**E**) biotinylated F8T2 in *p*-Xylene. (**F**) Avidin in water. (**G**) BSA in water (up: UV light off; down: UV light on).

In this research, we also investigated the optical properties of the biotinylated F8T2 semiconductor in an organic solvent, such as *p*-xylene, methanol and dioxane (Figure 6). The biotinylated F8T2 polymer solutions in *p*-xylene itself showed intensive fluorescence with yellow-green color under 365 nm UV light, but without UV light the solution showed yellow color without fluorescence. After treatment with the BSA solution, the biotinylated F8T2 solution had hardly changed color without UV light and the fluorescence changed slightly from yellow to greenish-yellow under 365 nm UV light. In comparison with this, the fluorescent color of the solution, which was treated with the avidin solution, shifted remarkably from yellow/green to blue in dioxane and in methanol. The two different kinds of organic solvents, methanol and dioxane, had no remarkable influence on fluorescence effects. As a reference of the fluorescence change, the BSA and avidin solution alone indicated no fluorescence under 365 nm UV light.

Therefore, not only the biotinylated F8T2 polymer semiconductor but also any other organic semiconductor with biotin-functional groups in its side chains seems to be a possible candidate for the selective detection of biomolecules in solution-based processes. Especially the changes of optical properties of organic semiconductors, for example, the changes of fluorescence color under UV light in organic solvent may one of the important characteristics for the selective sensing of special bioactive materials in solution. It might also make possible the real time monitoring of sensing materials in fluids.

## 4. Conclusions

In conclusion, we report in this paper biotinylated semiconducting polymers based on fluorene and bithiophene co-polymer F8T2. These are partially biotinylated after the esterification of the biotin with corresponding alcohol-groups on the side chain in F8T2. The potential application of these polymers to an organic thin film transistor used as a biosensor is also presented. Their properties as an organic semiconductor were found to show typical *p*-type semiconductor curves. The functionality of this biosensor in the sensing of biologically active molecules such as avidin in comparison with BSA was established through a selective decrease in the conductivity of the transistor. Changes to the optical properties of this polymer were also investigated through the change of the UV-florescence color before and after treatment with avidin or BSA buffer solution.
